# Deciphering the role of *Paenibacillus *strain Q8 in the organic matter recycling in the acid mine drainage of Carnoulès

**DOI:** 10.1186/1475-2859-11-16

**Published:** 2012-02-03

**Authors:** François Delavat, Vincent Phalip, Anne Forster, Marie-Claire Lett, Didier Lièvremont

**Affiliations:** 1 Génétique Moléculaire, Génomique, Microbiologie, UMR7156 Université de Strasbourg/CNRS, Strasbourg, France; 2 Laboratoire d'Ingénierie des Polymères pour les Hautes Technologies, EAC4379 ECPM-Université de Strasbourg, Strasbourg, France; 3 Génétique Moléculaire, Génomique et Microbiologie, Institut de Botanique, Université de Strasbourg, 28 rue Goethe, Strasbourg 67000, France

**Keywords:** *Paenibacillus*, Functional redundancy, Acid Mine Drainage (AMD), Amylase, Xylanase, Polymer degradation, Organic matter, Function-based screening, Community function

## Abstract

**Background:**

The recycling of the organic matter is a crucial function in any environment, especially in oligotrophic environments such as Acid Mine Drainages (AMDs). Polymer-degrading bacteria might play an important role in such ecosystem, at least by releasing by-products useful for the rest of the community. In this study, physiological, molecular and biochemical experiments were performed to decipher the role of a *Paenibacillus *strain isolated from the sediment of Carnoulès AMD.

**Results:**

Even though *Paenibacillus *sp. strain Q8 was isolated from an oligotrophic AMD showing an acidic pH, it developed under both acidic and alkaline conditions and showed a heterotrophic metabolism based on the utilization of a broad range of organic compounds. It resisted to numerous metallic stresses, particularly high arsenite (As(III)) concentrations (> 1,800 mg/L). Q8 was also able to efficiently degrade polymers such as cellulose, xylan and starch. Function-based screening of a Q8 DNA-library allowed the detection of 15 clones with starch-degrading activity and 3 clones with xylan-degrading activity. One clone positive for starch degradation carried a single gene encoding a "protein of unknown function". Amylolytic and xylanolytic activities were measured both in growing cells and with acellular extracts of Q8. The results showed the ability of Q8 to degrade both polymers under a broad pH range and high As(III) and As(V) concentrations. Activity measurements allowed to point out the constitutive expression of the amylase genes and the mainly inducible expression of the xylanase genes. PACE demonstrated the endo-acting activity of the amylases and the exo-acting activity of the xylanases.

**Conclusions:**

AMDs have been studied for years especially with regard to interactions between bacteria and the inorganic compartment hosting them. To date, no study reported the role of microorganisms in the recycling of the organic matter. The present work suggests that the strain Q8 might play an important role in the community by recycling the scarce organic matter (cellulose, hemicellulose, starch...), especially when the conditions change. Furthermore, function-based screening of a Q8 DNA library allowed to assign an amylolytic function to a gene previously unknown. AMDs could be considered as a reservoir of genes with potential biotechnological properties.

## Background

Ecosystems are complex systems driven by two major forces: a biotic community and abiotic conditions. The two forces interact with each other in a dependent manner. Physicochemical characteristics condition both the presence and relative abundance of each species, whereas the community modifies and adapts its surrounding environment to survive and grow. In these complex ecosystems, bacteria play key roles as they are able to efficiently change the environment, at the local scale (μm scale) as well as at much larger scales (earth scale). For example, *Cyanobacteria *are responsible for the major event that transformed the biogeochemistry of the earth about 3 billion years ago that is oxygen-evolving photosynthesis [1,2]. Many studies highlighted the role of bacteria in the transformation of the abiotic conditions, such as anaerobic ammonium oxidation [3], oxidation of arsenite and/or iron followed by a co-precipitation of these inorganic elements [4], or degradation of pollutants such as naphtalene [5]. All these processes are catalysed by bacteria but the corresponding activities are driven by their environment.

Polymers degradation by bacteria is also driven by the *in situ *conditions. Recently, it has been shown that cellulose degradation in peat extracts (pH 4.0) was significantly enhanced by adding nitrogen [6]. Furthermore, hydrolysis of polymers is of great interest for industries as it can be used in a wide range of applications, from paper industries to biofuel production [7]. For these reasons and because of the development of metagenomic approaches, many studies focused on the screening for new polymer-degrading enzymes [8-10]. Therefore, function-based screening of a DNA library allowed the discovery of various enzymes such as amylases, cellulases and lipases. Furthermore, function-based screening allows the detection of genes, without *a priori *and could therefore lead to the discovery of completely new sequences.

The Acid Mine Drainage (AMD) of Carnoulès (France) is an extreme environment characterized by very acidic conditions (pH 2.7-3.4) and heavy arsenic and iron contaminations in the water (up to 350 mg/L and 2,700 mg/L respectively) [11]. This oligotrophic environment is however not devoid of life as bacterial communities are active *in situ *[12]. In AMDs, many studies focused on the role of both cultured and uncultured bacteria in the transformation of inorganic compounds such as the oxidation of iron or arsenic, their resistance to metals and their ability to grow at low pH [4,11,13-16]. However, to our knowledge and despite its importance in any ecosystem, no study reported the role of bacteria in the recycling of organic matter in such oligotrophic environments.

In this study, a strain belonging to the genus *Paenibacillus *was isolated. As *Paenibacillus *species are known for the number of secreted enzymes [17-20] and because they were never described in AMDs, physiological, biochemical and molecular biology experiments were implemented to decipher the role of the strain Q8 in this extreme environment. Polymer-degrading activities of this bacterium were focused, both in homologous and heterologous conditions showing that even if this bacterium was isolated from a very acidic environment, this strain was still alive and active at neutral and alkaline conditions as Q8 was also able to degrade carboxymethylcellulose, hemicellulose (xylan) and starch in these conditions. A functional screening of a Q8 DNA-library in *Escherichia coli *allowed the detection of 15 clones positive for starch degradation and 3 for xylan degradation. Interestingly, the amino acids sequence of one starch-degrading enzyme was not identified *in silico *as an amylase, and corresponded to a "protein of unknown function". By its polymer-degrading activities, this strain may be important in the community function at least by providing easily degradable by-products to the rest of the community, contributing therefore to the functional redundancy.

## Results

### Growth characteristics with respect to the *in situ *conditions

**Figure 1:**
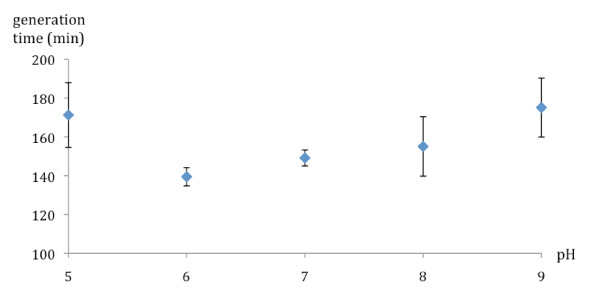
**Growth rate of *Paenibacillus *Q8 in LB medium at different pH**. Blue lozenges represent the generation time of Q8. Growth experiments were done at least in quadruplicate in LB medium at 30°C. As no growth occurred under pH 5 and above pH 9, the corresponding generation times are not reported in the figure.

*Paenibacillus *sp. strain Q8 was isolated from the soft unstable layer corresponding to the 1-2 cm of particles sedimenting on the bottom of the water stream. The samples were collected directly below the running water of Carnoulès characterized by very acidic conditions (pH 3.2) and by heavy metals and arsenic contamination (Table 1). In order to test whether this strain has adapted to the extreme conditions found *in situ*, its growth rate in LB medium under a broad range of pH *i.e*. from 3 to 10 was measured. Q8 was able of rapid growth under a broad range of pH, from 6 to 8 without significant variation in the generation time (≈140 min^-1^) (Figure 1). Moreover, the strain was able to grow under both acidic (pH 5) and alkaline (pH 9) conditions with a generation time only slightly higher (*e.g*. ≈175 min^-1 ^at pH 9). Additionally, no growth occurred for Q8 at pH 10 (data not shown). According to our data, Q8 is not a true acidophilic bacterium, as its growth was completely inhibited at pH below 5 (data not shown).

**Table 1:** Comparison of the metal resistance of Q8 with the *in situ *metals concentrations

Metal	Resistance to (mg/L)	*In situ *concentration (mg/L)
Ni	50	0.86

Cu	> 250	0.28

As(III)	> 1,800	161.4

Mn	200	11.82

Hg	1	ND

As(V)	> 5,000	33.37

Ag	> 10	ND

Cr(III)	> 50	0.10*

ND = Not Determined. *No speciation for chromium was undertaken. Observations were done after 72 hours incubation in LB medium supplemented with the corresponding metal.

To further decipher the adaptation of Q8 to the *in situ *conditions, its resistance for various metals at different concentrations was tested. As shown in Table 1, this strain was able to grow under high metal stresses. For instance, Q8 was still able to grow at the concentration of 1800 mg/L of As(III), which is by far much higher than the *in situ *As(III) concentration of Carnoulès water at the Q8 sampling date (161.4 mg/L). In addition to metal resistance, Q8 was tested for its capability to resist to various antimicrobial agents. On solid media, the strain was resistant to chloramphenicol, gentamycin, kanamycin, streptomycin, spectinomycin and trimethoprim and not to ampicillin, nalidixic acid, rifampicin and tetracycline. All these results further pointed out its resistance to various stresses and its high adaptation capabilities.

The catabolic capabilities of Q8 were tested and this showed that this bacterium used a broad range of organic compounds, including sugars monomers (glucose, fructose, lactose...) as well as various polymers, such as pectin, starch and glycogen (Additional file 1). The Q8 ability to degrade polymers has then been tested on LB plates supplemented by cellulose (in its soluble form Carboxymethylcellulose CMC), hemicellulose (in the form of its major compound *i.e*. xylan) and starch. This screening allowed to confirm the starch-degrading activity already detected with API 50 CH. Moreover, Q8 was shown to degrade CMC and xylan after 24 hours incubation, thus demonstrating two more polymer-degrading activities (data not shown).

### From phenotypic to genotypic characterization

To characterize the genes involved in the degradation of the different polymers, a DNA library in the pcDNA2.1 vector was constructed and screened for cellulose (CMC), hemicellulose (xylan) and starch degradation capabilities. The size of the fragments was 1-5 kb in average. Among the 6700 clones screened for the 3 activities, 15 displayed hydrolytic activity on LB plates supplemented with 1% soluble starch that represented 0.22% of the total number of clones. Three (0.045%) were positive on LB plates supplemented with 0.2% xylan (data not shown). No positive clone was obtained on LB plates supplemented with 0.2% CMC for the degradation of cellulose.

Two clones positive for starch degradation (BB9 and KF4) and one for xylan degradation (GαC5) were randomly chosen and the corresponding inserts were sequenced. The sequences were analyzed using the ORF Finder program from the NCBI (http://www.ncbi.nlm.nih.gov/projects/gorf/) and the corresponding ORFs were investigated with the NCBI-nr BLAST program.

Clone BB9 (2,152 bp), which was positive for the degradation of starch, carries a single ORF (1,614 bp) with 84% and 93% sequence identity at the nucleotidic (X60779) and proteic (CAA43194) level respectively, with the α-amylase gene *amyE *from *Bacillus circulans*.

Clone KF4 (1,635 bp) carries only one complete ORF (900 bp) whereas the two other possible ones were only 309 and 291 bp in length, and were therefore discarded. The 900 bp ORF encoded a hypothetical 299-aminoacids protein with unknown function, only partly related to a putative uncharacterized protein of *Paenibacillus polymyxa *(42% identity with YP 003869263, the first hit found by BLASTp search). The protein sequence was analyzed using the BLAST search at the Uniprot website (http://www.uniprot.org) and putative domains were searched using the prosite (http://prosite.expasy.org/) and InterProScan. Interestingly, this ORF showed no conserved hydrolytic domain, and did not share any sequence homology with proteins belonging to the different glycoside hydrolase (GH-) families.

Lastly, an ORF found in the clone GαC5 (867 bp, 288 aa) shared 76% nucleotidic identity (at a query coverage of 93%) with a predicted glycosidase of *Paenibacillus polymyxa *E681 (CP000154) and 80% at the proteic level (query coverage 95%) with a glycosidase-like protein of *Paenibacillus polymyxa *SC2 (YP003949339). This ORF presented domains that allowed the classification of the corresponding protein in the glycosyl hydrolase families GH43, 62, 32 and 68. The family GH-43 includes proteins known to degrade xylan (EC 3.2.1.8) (http://www.cazy.org).

### Partial characterization of the polymer-degrading activities

In order to characterize the global starch- and xylan-degrading activities of Q8, polymer degradation experiments were performed, both with whole cells and with crude extracts of *Paenibacillus *sp. strain Q8.

**Figure 2:**
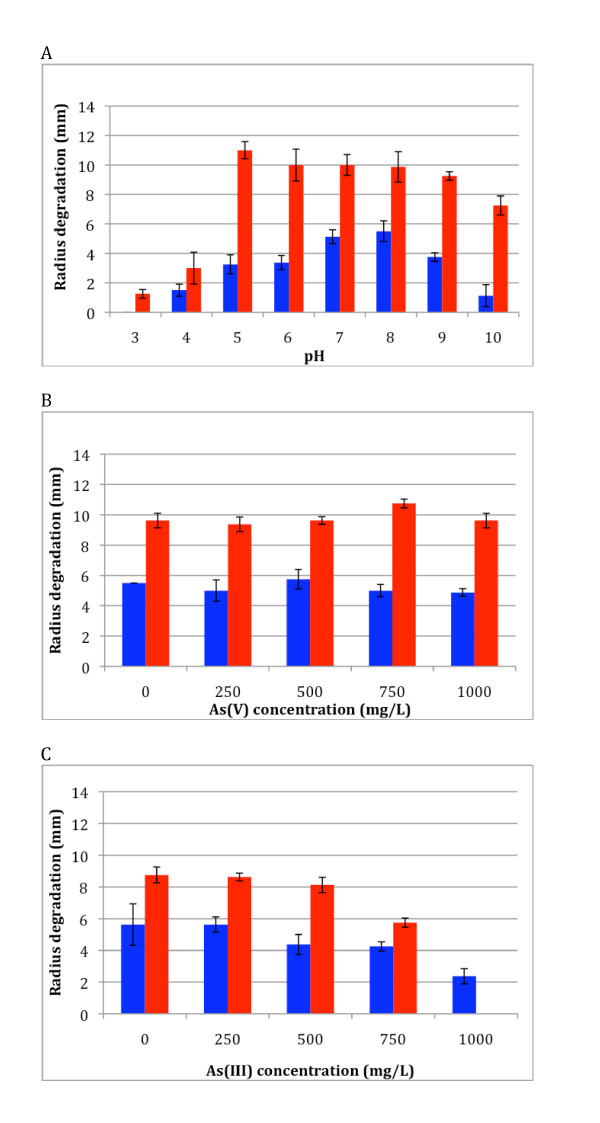
**Polymer degradation activities of *Paenibacillus *Q8 on Petri plates as a function of different stresses**. 2A: as a function of pH, 2B: as a function of As(V) concentration and 2 C: as a function of As(III) concentration. Activities were measured on LB plates supplemented with either 0.2% xylan or 1% starch and read after 34 h incubation. In red: xylan degradation. In blue, starch degradation.

After 34 hours incubation on LB plates supplemented with either starch or xylan, degradation halos were measured at different pH and arsenic (either arsenite As(III) or arsenate As(V)) concentrations. Q8 was able to efficiently degrade both polymers at pH 5-9 (Figure 2A). Furthermore, despite the absence of visible growth in liquid medium below pH 5 and above pH 9 during the course of experiments, Q8 was able to degrade both polymers at pH 4 and 10, and xylan at pH 3, albeit at lower efficiency (*e.g*. more than five fold decrease for xylanolytic activity at pH 3 as compared to pH 5-8). An increase of the As(V) concentration up to 1,000 mg/L had no effect on the polymer-degrading activities of Q8 (Figure 2B). These results confirmed also the resistance of Q8 to high As(V) concentration. In contrast, although Q8 was able to grow at 1,000 mg/L As(III) (Table 1), such concentration inhibited completely the xylanolytic activity of the strain (Figure 2C). Thus, the presence of As(III) seems to have a much higher impact on the xylan-degrading activity than on the starch-degrading activity.

**Figure 3:**
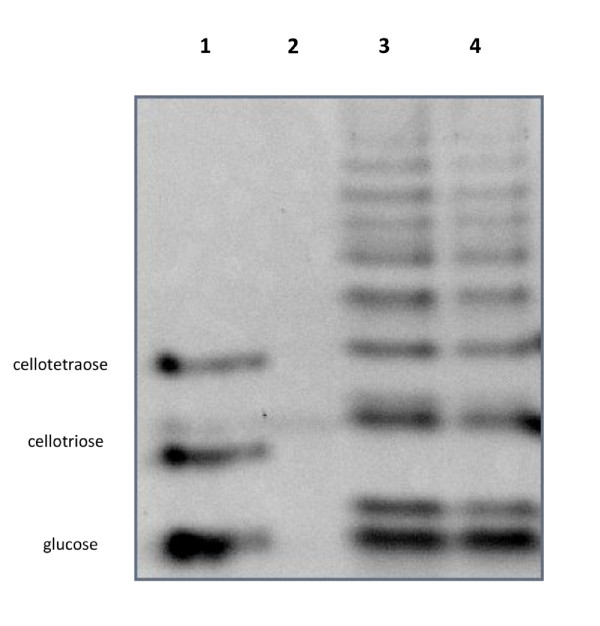
**Analysis of the products formed by *Paenibacillus *Q8 crude extracts with starch as substrate**. Lane 1: the standards cellotetraose, cellotriose and glucose (from top to bottom), lane 2: untreated starch, lane 3: substrate digested with the crude extract following the growth of the bacteria on LB and lane 4: substrate digested with the crude extract following the growth of the bacteria on LB-Starch.

To further decipher the polymer-degrading activity, *in vitro *experiments with the crude extracts of Q8 grown in liquid LB medium, supplemented or not with either xylan or starch, were performed. Starch degradation was monitored by the measurement of the glucose formed after the incubation of the crude extracts with starch. For xylan degradation, reaction was monitored by the measurement of reducing sugars. Lastly, *in vitro *activity was measured both at pH 7 (pH of the LB medium) and at pH 3 (close to the pH measured *in situ*). When using starch as substrate for the crude extracts obtained for Q8 grown on LB and in LB-starch, a low activity was detected at pH 7 (5.21 +/-0.44 and 1.13 +/-0.36 nmol/h.mg of protein, respectively, Figure 3). These activities were globally within the same order of magnitude, indicating that a large part of these enzymes are probably expressed constitutively. Polysaccharide Analysis using Carbohydrate gel Electrophoresis (PACE) experiments confirmed the *in vitro *activity of the crude extracts. Indeed, bands comigrating with glucose were clearly observed at the bottom of the gel (Figure 3) for both starch-induced and non-induced cells. Furthermore, numerous other bands corresponding to glucose oligosaccharides were also formed. It should be noticed that the intensity of the glucose band is not particularly higher than the intensities of oligomers. This means that the crude extract contained mainly endo-acting amylases. Lastly, no starch-degrading activity was measured at pH 3 for the two crude extracts (data not shown). This is in good accordance with the results obtained on solid media (Figure 1A).

**Figure 4:**
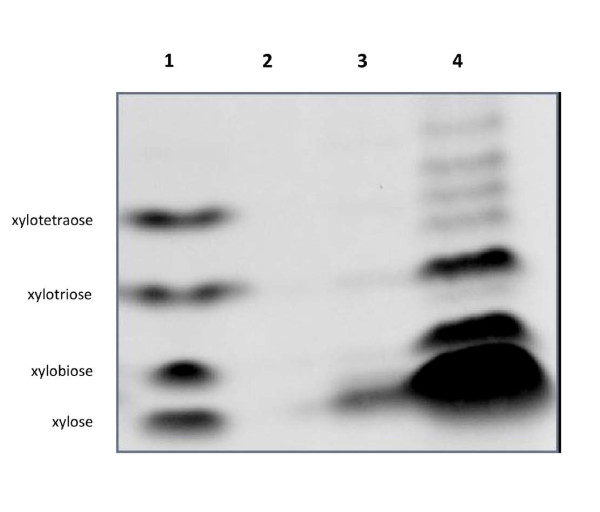
**Analysis of the product formed by *Paenibacillus *Q8 crude extracts with xylan as substrate**. Lane 1: the standards xylotetraose, xylotriose, xylobiose and xylose (from top to bottom), lanes 2: untreated xylan, lane 3: substrate digested with the crude extract following the growth of the bacteria on LB and lane 4: substrate digested with the crude extract following the growth of the bacteria on LB-Xylan.

High xylanase activity (2.09 +/-0.27 μmol/h.mg of protein) was detected when the cells were grown in the presence of xylan (Figure 4). In contrast, only a low activity (0.06 +/-0.02 μmol/h.mg of protein) was measured without induction of the cells with xylan, indicating that the xylan-degrading activity was mainly inducible. PACE experiments confirmed this hypothesis and showed that after induction with xylan, the bacteria produced enzymes able to yield an huge band of xylose and also small oligomers of xylose in much smaller quantities. This demonstrates that both endo-and exo-degrading activities were present with prevalence for the latter one. As for starch, no xylan-degrading activity was measured at pH 3.

## Discussion

Bacteria belonging to the genus *Paenibacillus *are widespread in various terrestrial environments. This genus is also known for its important enzymatic arsenal [21]. As such, those bacteria are able to interact with their surrounding environment and to transform it. This strategy has an important impact, both for *Paenibacillus *bacteria and for the microbial community as a whole. However, despite its presence and activity in various soils, this genus had never been detected in extreme environments such as AMDs, neither by culture-dependent nor -independent approaches before the isolation of Q8. To our knowledge, only a very recent work hypothesized the occurrence of a *Paenibacillus macerans *strain in AMDs [22]. In this last study, the use of GeoChip permitted to detect the presence of functional genes and a *nifH *gene close to the one of *Paenibacillus macerans*. However, this last bacterium was not detected *per se*, neither by culture nor by using a 16S rDNA library.

Q8 was isolated from the sediment of the Carnoulès AMD, characterized by very acidic (2.7-3.5) conditions and by heavy metals and arsenic contamination [11]. In order to decipher its role(s) in the community, the growth of the bacterium was tested under various pH conditions and in presence of metals or antibiotics. Although originated from an acidic environment, Q8 was able to grow under both acidic (pH 5) and alkaline (pH 9) conditions (Figure 1). It is noteworthy that under the tested conditions (LB medium at 30°C), the strain was unable to grow at pH below 5. This observation can be linked to the fact that moderate acidophilic bacteria have previously been isolated from AMDs [16]. Alternatively, the ability of Q8 to grow at alkaline pH but not at very acidic pH may be related to the *in situ *occurrence of micro and macro-niches. Indeed, physical and chemical conditions can vary between two niches separated physically by a millimetre or centimetre order of magnitude [23-25]. It is therefore likely that *Paenibacillus *species live *in situ *in micro-environments where the pH is less acidic than the global pH measured in the ecosystem. It should be noted that this observation was confirmed by the isolation from the Carnoulès site of alkaliphilic bacteria, belonging to the genera *Micromonospora *and *Thiomonas*, using solid media at pH 8 and 9.8, respectively (unpublished work).

Metal and arsenic concentrations can vary at millimetre scale, but also during the course of the year [26] and the bacteria living in Carnoulès have to adapt to these extreme variations. The resistance of Q8 to high concentration of various metals (Table 1) would therefore allow *Paenibacillus *species to grow and to be active under such stresses. Arsenic stress is one of the most important stresses in Carnoulès, as exceptionally high concentration (up to 350 mg/L) in the water. A MIC of 750 mg/L As(III) (10 mM) was observed in a previous work for *Thiomonas *sp. 3As also isolated from Carnoulès [27]. The present work showed that Q8 was still able to grow with an As(III) concentration twice as high as for *Thiomonas *sp. 3As (Table 1).

Taken together, the high growth rate measured for Q8 (Figure 1) and the high metal resistance (Table 1) highlighted its possible ability to adapt to drastic physical and chemical changes in its environment, such as an increase of the pH or the metal concentration. At the ecosystem level, bacteria that can grow under less acidic or even alkaline conditions can play a major role in the resilience of the community, *i.e*. the capability of the community to adapt to physico-chemical changes in the environment [28]. This resilience is dependent on 1) the community structure, comprising both the abundant bacteria and the rare biosphere [29] and 2) the genetic potential carried by both abundant and rare bacteria.

Here, the possible role(s) of Q8 in the community function was investigated. The Carnoulès site is considered to be at least a partly oligotrophic environment [4]. The ability to live in oligotrophic environments requires for every bacterium specific adaptations to find essential nutrients, especially in terms of carbon sources (for review see [30]). This necessity is further amplified when additional stresses occur, such as metal and acidic stress. Different strategies are possible in such environments. For example, it has been recently shown that most bacteria isolated from the AMD of Carnoulès were able to grow on FD2 medium, characterized by very low carbon concentration (0.01% casaminoacid) (unpublished work). Others were able to use inorganic carbon (autotrophic growth), as does *Thiomonas arsenivorans*, which was also able to use the oxidation of As(III) as sole energy source [31]. Another adaptation concerns the capability carried by some strains to metabolize unusual carbon compounds. These compounds can be pollutants (eventually toxic for other strains) such as naphtalene [5], or polymers.

In our study, Q8 was able to use various carbon sources, and to efficiently and rapidly degrade starch, cellulose and hemicellulose (xylan). The degradation of monomers is not surprising, since the majority of the bacteria possess the metabolic pathways to use glucose or fructose. However, the degradation of various polymers is more surprising when considering the *in situ *conditions. Moreover, the starch and xylan degradation activities were more important when decreasing the acidic stress (Figure 2A), confirming that the growth depends on the pH. Furthermore, its ability to degrade xylane and starch was not affected by the presence of up to 1,000 mg/L As(V) (Figure 2B). The increase of the As(III) concentration led to a decrease of the polymers degradation activities, but activity was still measured with 750 mg/L As(III) (Figure 2C). The *in vitro *polymer degradation using the acellular extracts confirmed the activity at pH 7 and absence of activity at pH 3. Moreover, the gene expression of the amylases was constitutive and corresponded to both endo- and exo-activities, whereas xylan degradation required an induction of the corresponding genes to perform the mainly exo- but also endo-degradation of xylan.

Function-based screening allowed the characterization of an amylase (BB9) and a xylanase (GαC5). Moreover, this strategy allowed us to assign an amylolytic function to a gene previously identified as encoding a "protein of unknown function" (KF4), since the protein sequence showed no sequence homology with any already described amylase or glycosidase, nor possessed any known hydrolytic domain. This result can be compared to the one obtained by Graham *et al. *[32], who described a novel cellulase in an *archaea *consortium originated from a geothermal source (temperature 90°C). This enzyme had an optimum activity at 109°C, and possessed 2 domains that were not yet observed in hyperthermophilic cellulases. However, since the authors have used a sequence-based approach to find the corresponding gene, the aminoacid sequence of the novel cellulase is therefore not be completely different from others. Moreover, a screening for amylases using metagenomic DNA from an AMD led to the detection of two amylases sharing no significant proteic sequence similarity with any known amylase or glycosidase. Purification and characterization confirmed the amylolytic function carried by both proteins (Delavat *et al*., submitted). One possible explanation for this novelty is that the extreme conditions found in Carnoulès (in terms of pH and metal concentrations) would trigger an accelerated adaptation for many important bacterial processes, such as polymers degradation.

Therefore, Q8 presents a heterotrophic metabolism and may be able to change its surrounding environment by degrading complex polymers. A recent study based on functional gene array (GeoChip 2.0) in an AMD in China allowed the detection of genes involved in the degradation of cellulose, lignin and chitin [22]. However, this method is based on the detection of labeled DNA, which represents the metabolic potential found in the community, but no insights was made into the functional activity as no activity was detected.

To our knowledge, no study focused on the role of a cultured organism isolated from AMDs in the recycling of complex organic matter. A recent meta- and proteogenomic study allowed the reconstruction of 7 genomes in Carnoulès, corresponding to the 7 dominant bacteria, called CARN1 to CARN7 [4]. In this previous study, it was shown that CARN6 was the sole bacterium carrying the genes involved in cellulose metabolism. In depth analysis of the 7 genomes (http://www.genoscope.cns.fr/agc/microscope/carnoulescopes) showed that genes encoding xylanase were only found in CARN3, and genes encoding amylases were found in CARN3, CARN5 and CARN6. However, any ecosystem needs recyclers that transform complex organic matter, and, moreover, any ecosystem needs functions redundancy to assure the corresponding function when the conditions change *i.e*. when the relative abundance of the bacteria change. Here, *Paenibacillus *sp. strain Q8 was able of cellulolytic, hemicellulolytic and amylolytic activities, especially when the acidic conditions were less stringent. The bacterium Q8 is likely to use complex carbon compounds in the AMD of Carnoulès, where they are scarce. It may therefore beneficiate of this extra source, on the contrary to many other microorganisms from the same site. At the community level, the strain Q8 can play a major role in the recycling of organic molecules by degrading complex compounds, especially when the conditions change.

## Conclusion

*Paenibacillus *sp. strain Q8 was isolated from the arsenic- and metal-contaminated AMD of Carnoulès (France). This strain was able of rapid growth under both acidic and alkaline conditions, and was resistant to high metal concentrations, highlighting its ability to survive under the extreme conditions found *in situ*. This strain carried an arsenal of polymer-degrading enzymes, as it degraded starch, cellulose and hemicellulose, and presented a polymer-degrading activity under a broad pH range and under high arsenic concentrations. Function-based screening of the Q8-DNA allowed the detection of amylases and xylanases. The genetic analysis of some clones allowed the characterization of a gene that was previously not assigned to any function. Therefore, function-based screening also allowed to assign a function to a gene and to further decipher the community function. At the community level, Q8 can act as a recycler of the organic matter by releasing by-products from complex polymers, in this oligotrophic site, to the whole community. This activity is particularly important when the *in situ *physico-chemical conditions change (*e.g*. in terms of pH) as the polymer-degrading activities for Q8 were more important under less acidic conditions. Therefore the strain Q8 participates to the community function, especially for the functional redundancy, hence for the resilience of the community.

To our knowledge, this is the first study reporting the polymer-degrading activity at the phenotypic level and the role of polymer-degrading bacteria in the community function in AMDs. This study highlights the complexity of the community function in the AMD of Carnoulès. Further work has to be undertaken to understand the role of other bacteria in the community function. More in depth analysis of the *in vitro *polymer-degrading activities would also provide interesting informations, both in fundamental biology and in biotechnology for the finding of proteins with specific characteristics with regards to the *in situ *conditions.

## Methods

### Chemical analysis

Chemical analyses were performed on the Carnoulès water running above the soft unstable sediment collected for the isolation of Q8 according to procedures described previously [33,34].

### Bacterial strains, plasmids and growth conditions

*Paenibacillus *sp. strain Q8 was isolated from the sediment (up to 2 cm deep) of the Carnoulès AMD (France) using the Soil Substrate Membrane System (SSMS) as described by Ferrari *et al. *[35]. Briefly, sediment sample taken from the same site was used as a growth medium, and 1 mL of a 1:100 dilution sample served as bacterial inoculum. After the incubation time (10 days at 20°C), the membrane was removed, cut with a sterile razor blade and vortexed 1 min with 1 mL 0.9% NaCl and 100 μL of the supernatant was then spread onto LB plates. This strain was routinely grown in LB medium at 30°C unless otherwise stated.

*Escherichia coli *strain DH5α (Invitrogen) was used for the heterologous expression of the genes from *Paenibacillus *sp. Q8. Vector pcDNA2.1 was used as cloning vectors for the DNA library construction. *E. coli *strains were routinely grown on LB agar (MP Biomedicals) plates supplemented by 100 mg/L ampicillin and incubated at 37°C.

### Physiological properties of *Paenibacillus *sp. Q8

Growth at different pH was monitored by measuring the absorbance (600 nm) in LB medium adjusted to pH 3-10 (increment 1) with H_2_SO_4 _and KOH before autoclaving.

*Paenibacillus *resistance to each metal was investigated in LB broth supplemented with the respective metal at different concentrations from stock solution previously adjusted to pH 7 and filtered-sterilized. Metals were provided in the form of salts as follows: C_4_H_6_**Ni**O_3_,4H_2_0; **Cu**SO_4,_5H_2_0; Na_2_H**As**O_4_,7H_2_0; Na**As**O_2_; **Mn**SO_4_; **Cr**Cl_3,_6H_2_0; **Hg**Cl_2_; **Ag**NO_3_. Stock solutions were conserved at 4°C until use.

Antibiotic resistance tests were performed using the disc method. Antibiotics and concentrations were as follows: Nalidixic acid (1.5 μg/disc), Ampicillin (3 μg/disc), Chloramphenicol (0.6 μg/disc), Gentamycin (0.06 μg/disc), Kanamycin (0.6 μg/disc), Rifampicin (7.5 μg/disc), Streptomycin (0.6 μg/disc), Spectinomycin (1.5 μg/disc), Tetracycline (0.24 μg/disc), and Trimethoprim (0.15 μg/disc).

The GEN III Microplate™ (Biolog) were used (with the fluid IF-A) and API 50 CH (with the API 50 CHB/E medium) (Biomérieux) to screen for organic molecule degradation, according to the manufacturer's protocol.

All experiments were done at least in quadruplicate.

### Polymer-degrading activity

*Paenibacillus *sp. strain Q8 and the DNA library were screened for the degradation of different polymers. Amylolytic activity was first screened on LB plates supplemented with 1% starch (Sigma). After 24 hours, the plates were flooded with lugol solution (Sigma) and a clear halo around the colonies indicated degradation of starch. Hemicellulolytic activity was detected by the apparition of a clear halo around the colony upon flooding with lugol solution. Cellulolytic activity was detected on LB plates supplemented with 0.2% Carboxymethylcelullose CMC (Sigma). Plates were stained with Congo red (0.2%) for 20 min and plates were washed with 1 M NaCl. Cellulase-expressing colonies were surrounding by a yellow halo.

For polymer degradation activity as a function of pH, LB medium supplemented with the corresponding substrate was adjusted to pH 3-10 with KOH and NaOH (6 M) prior to sterilization by autoclaving. For polymer degradation activity as a function of As(III) and As(V) concentration, the metal was added and the pH adjusted to pH 7 before autoclaving. After deposing 10 μL of a Q8 suspension, the plates were incubated at 30°C for 34 hours before reading.

All experiments were done at least in quadruplicate.

### *In vitro *activity of the crude extracts of *Paenibacillus*

*Paenibacillus *sp. strain Q8 cells grown on LB medium (1 L) supplemented or not with either 1% wheat starch (Sigma, S2760) or 0.2% xylan from beechwood (Sigma, X4252) was collected by centrifugation (5,000 g, 10 min).

The pellets were resuspended in a minimal volume (1.5 to 2.5 mL) of Cellytic B (Sigma) containing 0.2 mg/mL of lysozyme, 48 U/mL of DNAseI and the appropriate volume of the anti-proteases cocktail Complete (Roche). They were incubated 30 min at 25°C under agitation. The supernatants, containing the bacterial crude extracts were then recovered after centrifugation (10,000 g, 30 min, 4°C). Protein concentrations were evaluated for each crude extract using the Bradford reagent (BioRad) and with BSA as standard. The action of the crude extracts on polysaccharides was tested by adding 350 mg of total proteins with a 1% solution of each substrate (potato and wheat starch, and birchwood xylan) prepared either in 0.1 M glycine-HCl buffer pH 3.0 or in 0.1 M phosphate buffer pH 7.0. Incubations were performed under agitation at 45°C and 100 μL samples were taken regularly and subsequently boiled for 10 min to stop enzymatic reaction. Glucose content was estimated using the chromogen 2,2'-azino-bis(3-ethyl benzthiazoline-6-sulfonate). Reducing sugars were measured by the dinitrosalicylic acid method using xylose as standard. Standard deviations were calculated from two determinations.

To visualize the oligosaccharides produced during the reaction, Polysaccharide Analysis using Carbohydrate Electrophoresis [36] was performed. Briefly 50 μL of the reaction mixture were dried, the products labelled with a fluorophore, thereafter separated by electrophoresis and visualized under UV.

### Construction of the DNA library

*Paenibacillus *Q8 DNA was extracted using the Wizard^® ^Genomic DNA Purification Kit (Promega) and nebulized for 36 seconds and DNA fragments (1-5 kb) were gel-purified (MP biomedicals). The purified fragments were repaired using the T4 DNA polymerase and *Bst*X I adapters (Invitrogen) were ligated to both ends. Plasmid pcDNA 2-1 and *Bst*X I-ligated Q8 DNA fragments were digested with *Bst*X I and ligated using a T4 DNA ligase (Fermentas). DH5α electrocompetent cells were transformed and white colonies were picked and stored in 96-well microplates at -80°C until use.

### Sequencing and analysis of the positive clones

Plasmid from positive clones were extracted with the QIAprep Spin Miniprep kit (Qiagen) and sequenced by Millegen with the universal primers M13(-21) (TGTAAAACGACGGCCAGT) and M13R(-29) (CAGGAAACAGCTATGACC). ORFs were searched using the ORF Finder program at the NCBI website ORFs sequence analyses were performed by using the NCBI-nr BLAST program (http://blast.ncbi.nlm.nih.gov/) and the Uniprot website (http://www.uniprot.org). Putative domains were searched using the prosite (http://prosite.expasy.org/). The sequences of clones BB9, KF4 and GαC5 were submitted to the EMBL databases under the accession numbers HE617173, HE617174 and HE617175, respectively.

## Competing interests

The authors declare that they have no competing interests.

## Authors' contributions

FD, VP, MCL and DL conceived, supervised and coordinated this study. FD carried out the physiological experiments, and the cloning, screening and sequencing steps. VP and AF carried out all *in vitro *biochemical experiments. FD and VP performed the interpretation of data. FD wrote the manuscript and VP, MCL and DL critically revised the manuscript. All authors read and approved the final manuscript.

## Supplementary Material

Additional file 1 **List of substrates used by *Paenibacillus *sp. strain Q8**. For substrate utilization, both API 50 CH (Biomérieux) and GEN III Microplate™ (Biolog) were used and read after 48 hours incubation. Click here for file
