# Supplementary material for: Deciphering the role of Paenibacillus strain Q8 in the organic matter recycling in the acid mine drainage of Carnoulès
Source: Microb Cell Fact. 2012 Feb 3;11:16. doi: 10.1186/1475-2859-11-16 (PMC3287962; doi:10.1186/1475-2859-11-16)
Supplement: Additional file 1 — List of substrates used by Paenibacillus sp. strain Q8. For substrate utilization, both API 50 CH (Biomérieux) and GEN III Microplate™ (Biolog) were used and read after 48 hours incubation. [file 1475-2859-11-16-S1.DOCX]

Substrates degraded by *Paenibacillus* sp. strain Q8 as observed by API 50 CH (Biomérieux) and Gen III microplate (Biolog):

**API 50 CH:**

Glycerol, ribose, methyl-βD-xylopyranoside, D-galactose, D-glucose, D-fructose, D-mannose, D-mannitol, methyl-αD-glucopyranoside, N-acetylglucosamine, amygdalin, arbutin, esculin, salicin, D-cellobiose, D-maltose, D-lactose, D-melibiose, D-saccharose, D-trehalose, D-raffinose, starch, glycogen, gentiobiose, D-turanose.

**Biolog Gen III Microplates:**

Dextrin, D-maltose, D-trehalose, D-cellobiose, D-gentiobiose, sucrose, D-turanose, stachyose, D-raffinose, α-D-lactose, D-melibiose, β-methyl-D-glucoside, D-salicin, N-acethyl-D-glucosamine, N-Acetyl-β-D-mannosamine, α-D-glucose, D-fructose, D-galactose, D-mannitol, myo-inositol, glycerol, pectin, D-gluconic acid, methyl pyruvate, acetoacetic acid, acetic acid.
